# Risk of secondary malignancy after radiotherapy for breast cancer: long-term follow-up of Japanese patients with breast cancer

**DOI:** 10.1007/s10549-022-06644-x

**Published:** 2022-07-04

**Authors:** Noriyuki Okonogi, Kumiko Karasawa, Yuki Nitta, Yasumasa Mori, Kazutoshi Murata, Masaru Wakatsuki, Hiroshi Tsuji

**Affiliations:** 1 QST Hospital, National Institutes for Quantum Science and Technology, 4-9-1 Anagawa, Inage-ku, Chiba City, Chiba 263-8555 Japan; 2 Department of Radiation Oncology, Tokyo Women’s Medical University School of Medicine, 8-1 Kawata-chou, Shinjuku, Tokyo 162-0054 Japan

**Keywords:** Breast Neoplasms, Radiotherapy, Neoplasms, Second Primary

## Abstract

**Purpose:**

There have been very few reports of secondary malignancies after breast cancer treatment in Asia, particularly in Japan. This study aimed to evaluate the risk of secondary malignancies after radiotherapy (RT) in Japanese breast cancer patients.

**Methods:**

This single-center retrospective study included patients who underwent RT between July 1961 and September 2006 for postoperative breast cancer. A total of 702 patients with a follow-up period of more than 5 years were analyzed. All malignancies observed at more than 5 years after the start of RT were defined as secondary malignancies. To calculate the relative risk (RR) of secondary malignancies, we applied data from the National Cancer Center in Japan.

**Results:**

The median observation period was 9.7 (interquartile range 7.1–18.2) years. The cumulative person-years of observation were 6879.4. The RR of contralateral breast cancer increased by 1.85-fold (95% confidence interval [CI] 1.05–3.26) among patients compared with that among the general population; however, the difference was not significant (p = 0.053). The RR of secondary malignancies other than breast cancer increased by 2.71-fold (95% CI 1.99–3.70, p < 0.001) among the patients compared with the general population. Even when only malignancies detected more than 10 years after RT were defined as secondary malignancies, the RR of secondary malignancies other than breast cancer was 1.91 (95% CI 1.33–2.73, p < 0.001).

**Conclusion:**

The incidence of secondary malignancies after RT may be somewhat higher in Japanese patients with breast cancer than in the general population.

## Introduction

Breast cancer is a common cancer in many countries. In Japan, approximately 94,000 patients were diagnosed with breast cancer, and more than 15,000 individuals died of breast cancer in 2021 [1]. Radiotherapy (RT) plays a vital role in the treatment strategy of breast cancer together with surgery and systemic treatments: specifically, its role is to prevent local and regional lymph node recurrences. The effect of RT on local control is observed at a constant rate, regardless of age, tumor factors, or concomitant systemic treatment, being more effective in patients with a higher risk of local recurrence [2]. Moreover, RT improves the survival rate after breast-conserving surgery regardless of the presence of axillary lymph node metastasis [2]. Therefore, it is highly recommended unless the patient is pregnant or has a specific genetic disorder [3, 4].

Breast cancer has a long-term prognosis if treated appropriately. Hence, proper management and prevention of adverse events are essential. Dermatitis, subcutaneous tissue inflammation, and pneumonia are well-known adverse events in the acute phase after RT [5, 6]. Meanwhile, late adverse events include upper extremity edema, cardiac disease, and secondary malignancies [7–9]. In particular, secondary malignancies after breast cancer treatment can be fatal. Therefore, secondary malignancies, including contralateral breast cancer and other malignancies, after breast cancer treatment have been studied. Taylor et al. reported that RT increased the relative risk (RR) of breast cancer in the contralateral breast by 1.20-fold (95% confidence interval [CI] 1.08–1.33) [8]. A meta-analysis conducted by the Early Breast Cancer Trialists’ Collaborative Group also reported that RT increased the RR of breast cancer in the contralateral breast by 1.18-fold [2]. RT for postoperative breast cancer is also associated with an increased risk of secondary malignancies other than breast cancer. Taylor et al. reported that RT increased the RR of secondary malignancies other than breast cancer to 1.23 (95% CI 1.12–1.36), and Grantzau and Overgaard also reported an increase in the RR to 1.22 (95% CI 1.06–1.41) [8, 9]. Thus, postoperative RT for breast cancer increases the risk of secondary malignancies, although to a small degree.

Calip et al. reported that the risk of secondary malignancies differs according to race and ethnicity [10]. They assessed the risk of secondary malignancy among breast cancer survivors for each case group defined by race/ethnicity (non-Hispanic White, Black, Hispanic, and Asian/Pacific Islander) and found that Black and Asian/Pacific Islander women had a higher risk of secondary malignancies. Terao et al. recently reported that the genomic locations of mutations in their respective hematopoietic clones differed significantly between Japanese and European individuals and that these differences predicted the relative rates of chronic lymphocytic leukemia (which is more common in Europeans) and T-cell leukemia (which is more common in Japanese individuals) in these populations [11]. Although their study focused on leukemia and not solid tumors, the findings suggest the need for more research on the differences in the risk of secondary malignancies among racial groups. However, there are still very few reports on secondary malignancies after breast cancer treatment in Asia, particularly in Japan. Hence, this study aimed to evaluate the risk of secondary malignancies after RT in Japanese patients with breast cancer.

## Materials and methods

### Study design

This single-center retrospective study included patients who underwent RT between July 1961 and September 2006 for postoperative breast cancer at the National Institutes for Quantum Science and Technology (QST) Hospital. All RT procedures were performed in accordance with the standards of care for each decade [12]. The RT equipment for each age group was as follows: Vickers-Armstrong 6 MeV (1963–1973), Mitsubishi ML-15M2 (1973–1985), Mitsubishi ML-10X (1985–1996), Varian Clinac2100C (1997–2005), and Varian Clinac21EX (2005–2006). All RTs involved X-rays alone or a combination of X-rays and electron beams. No patients received intensity-modulated RT (IMRT). Linear accelerators delivered all RTs with 6 MV or 4 MV X-rays and ≥ 6 MeV electrons. In terms of irradiation fields, there were mixed irradiation cases of the conserved breast, chest wall, supraclavicular fossa, or parasternal/intermammary lymph node area. One thousand six hundred twenty-one patients were included in the study, out of whom 702 that had a follow-up period of more than 5 years were analyzed. All malignancies observed at more than 5 years after the start of RT were defined as secondary malignancies. This retrospective study was approved by the Institutional Ethics Review Board (N21-015). The need for written informed consent was waived owing to the retrospective nature of the study. Instead, a document for an opt-out policy was uploaded to the webpage of the QST Hospital, which allowed any of the patients and their families to refuse to be included in the study.

### Data collection

We collected data on the patients’ age at the time of RT initiation, dose of RT, date of RT initiation, date of the second cancer diagnosis, date of death or the last visit from the QST database, and medical records. As previously reported, we confirmed the information on secondary malignancy by checking medical records, radiology reports, surgical records, and pathology reports [13]. Considering the appropriate incubation period, as reported by Grantzau et al., secondary malignancies were defined as all malignancies observed for the first time at 5 years after the initiation of RT [9]. Only those lesions with a histological type differing from that of the initial breast cancer, and with no evidence of recurrence, were classified as secondary malignancies.

The patients were followed-up every 6 months until 5 years after RT and once a year after that. Patients who did not undergo face-to-face follow-up were sent a yearly questionnaire with specific questions on breast cancer recurrence, adverse events after treatment, and secondary malignancy development. Additional information on secondary malignancies was gathered by consulting other doctors, hospitals, and patients or their families. With the approval of the Ministry of Justice, missing patient data were supplemented from the Japanese nationwide registry, including the date and cause of death. The observation period started at the date of initiation of RT for breast cancer and ended at the date of death or last visit.

### Data analysis

To calculate the RR of secondary malignancies in the cohort, we applied data from the National Cancer Center in Japan, which shows site-specific cancer incidence rates in the general female population in Japan [14]. The RR of secondary malignancies was calculated by comparing the incidence rate of cancer in the general population with that of secondary malignancies per person-year in the study cohort. For the cancer incidence rates in the general population, age-adjusted cancer incidence rates were used to eliminate the effect of increased cancer incidence rates with aging. The Mann–Whitney U test or Welch’s *t* test was employed for continuous variables and the chi-square test with Yates’ adjustment for nominal variables. The cumulative incidence rate of secondary malignancies was calculated using the Kaplan–Meier method. The log-rank test was used to compare the cumulative incidence rates. All statistical tests were two-sided, and all comparisons were considered statistically significant when the p-value was less than 0.05. The Statistical Package for the Social Sciences for Macintosh, version 27.0, (IBM Inc., Armonk, NY, USA) was used for all statistical analyses.

## Results

Among the 702 patients analyzed in the study, the median age at the start of RT was 51 years (interquartile range [IQR] 44–61). The median observation period was 9.7 (IQR 7.1–18.2) years (Table 1). The cumulative person-years of observation were 6879.4. Of the 702 patients, 60 secondary malignancies were observed in 57 patients. At the onset of secondary malignancies, the median years was 14.4 years (IQR 9.0–24.2). There was no notable bias in calendar years when RT was conducted. The sites and number of secondary malignancies are listed in Table 2.

**Table 1:** Patients’ characteristics for all analyzed subjects

Characteristics (n = 702)	Median (IQR) or number (%)
Age at irradiation, year; median (interquartile range)	51 (44–61)
Irradiated dose, Gy; median (interquartile range)	50.0 (50.0–51.0)
Follow-up period, year; median (interquartile range)	9.7 (7.1–18.2)
Period of the RT (calendar year)	
1961–1984	350 (49.9)
1985–2006	352 (50.1)

*IQR* interquartile range, *Gy* Gray, *RT* radiotherapy

**Table 2:** Summary of secondary malignancies

Characteristics	n
Number of patients with secondary malignancies	57 (8.1%)
Years at the onset of secondary malignancies, median (interquartile range)	14.4 (9.0–24.2)
Number of all duplicated malignancies	60* (8.5%)
Site of the malignancy	
Contralateral breast	13
Lung	9
Colon and rectum	9
Stomach	6
Uterus	5
Ovary	5
Thyroid	3
Pancreas	3
Esophagus	2
Bladder	2
Kidney	1
Tongue	1
Skin cancer	1

*Three patients had tertiary malignancies

Table 3 lists the RRs of secondary malignancies by sites after RT. The RR of all secondary malignancies was 2.463 (95% CI 1.877–3.231), which increased significantly over the general female population (p < 0.001). RT increased the RR of breast cancer in the contralateral breast by 1.85-fold (95% CI 1.05–3.26) among the patients compared with the general population, although no significant difference was observed (p = 0.053). The RR of secondary malignancies other than breast cancer increased by 2.71-fold (95% CI 1.99–3.70, p < 0.001) among the patients compared with the general population. Even when only malignancies detected at more than 10 years after RT were defined as secondary malignancies, the RR of secondary malignancies other than breast cancer was 1.91 (95% CI 1.33–2.73, p < 0.001).

**Table 3:** Risk of secondary malignancies by site after radiotherapy

	Observed number of malignancies (Of these, no. of pts within 10 years after RT)	Expected number of malignancies	Relative risk (95% CI)	p-value
All malignancies	60 (17)	24.357	2.46 (1.88–3.23)	< 0.001
Contralateral breast	13 (3)	7.037	1.85 (1.05–3.26)	0.053
All sites except contralateral breast	47 (14)	17.321	2.71 (1.99–3.70)	< 0.001
Lung	9 (0)	1.866	4.82 (2.31–10.07)	< 0.001
Colon including rectum	9 (4)	3.255	2.77 (1.38–5.56)	0.009
Stomach	6 (2)	1.825	3.29 (1.39–7.77)	0.016
Ovary	5 (2)	1.094	4.57 (1.74–12.02)	0.009
Thyroid	3 (3)	1.146	2.61 (0.82–8.35)	0.118
Pancreas	3 (1)	0.800	3.76 (1.14–12.44)	0.063
Esophagus	2 (0)	0.227	8.81 (1.81–42.99)	0.041
Bladder	2 (0)	0.205	9.69 (1.94–48.46)	0.036
Kidney	1 (1)	0.448	2.24 (0.36–14.03)	0.393
Tongue	1 (1)	0.352	2.85 (0.44–18.35)	0.334
Skin cancer	1 (0)	0.488	2.05 (0.33–12.74)	0.417

*no.* number, *pts* patients, *RT* radiotherapy, *CI* confidence interval

*Numbers in parentheses indicate the number of secondary malignancies observed less than 10 years after RT

In the analysis by site of secondary malignancy, the RRs of secondary lung, colonic, rectal, stomach, ovarian, esophageal, and bladder carcinomas increased significantly. The secondary malignancies of the lung, esophagus, and bladder all developed at 10 years after RT. All patients with secondary ovarian carcinoma were less than 50 years of age at the start of RT. No significant differences in the RRs were observed for secondary thyroid, pancreatic, kidney, tongue, and skin malignancies among the patients compared with those in the general population.

**Fig. 1:**
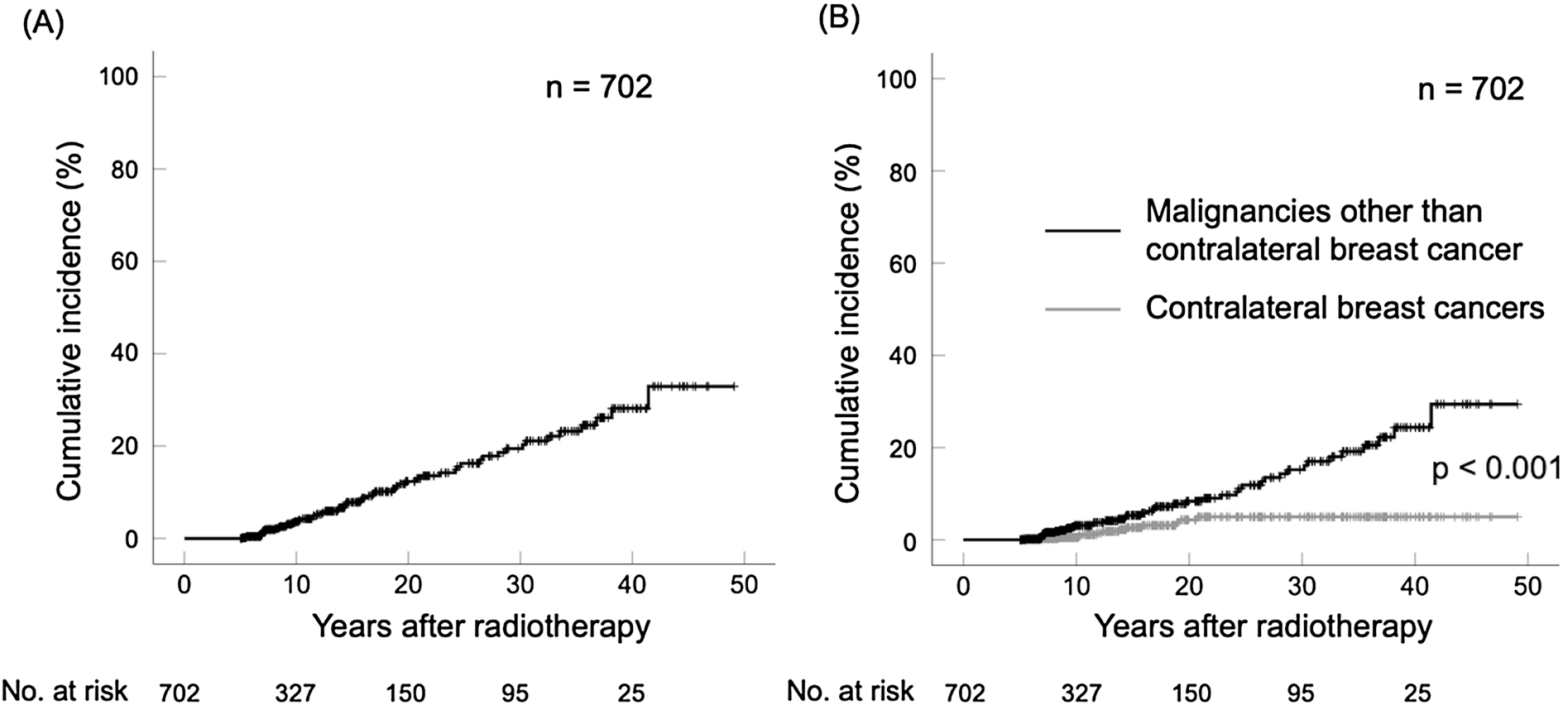
The incidence of **A** all secondary malignancies and **B** contralateral breast cancer and other secondary malignancies

The 10-year and 20-year cumulative incidence rates of secondary malignancies were 3.6% (95% CI 1.9–5.3) and 12.4% (95% CI 8.4–16.3), respectively. When age was dichotomized by median age (51 years), there was no significant association between age and the occurrence of secondary malignancies (p = 0.130). When the RT period was dichotomized (1961–1984 vs. 1984–2006), a higher incidence rate of secondary malignancies was observed in the group treated more recently (p < 0.001). The 10-year and 20-year cumulative incidence rates of contralateral breast cancer were 0.4% (95% CI 0.0–0.7) and 4.3% (95% CI 1.7–6.9), respectively. The 10-year and 20-year cumulative incidence rates of malignancies other than contralateral breast cancer were 3.1% (95% CI 1.5–4.7) and 8.4% (95% CI 5.1–11.7), respectively. Although contralateral breast cancer appeared by approximately 20 years after RT, the incidence rate of secondary malignancies other than contralateral breast cancer gradually increased over time. The incidence rate of contralateral breast cancer and other secondary malignancies were significantly different (p < 0.001) (Fig. 1).

## Discussion

To the best of our knowledge, this is the first long-term observational study on secondary malignancies after RT for breast cancer in Japan. Although this was a single-center study, it analyzed more than 700 cases and included an observational period of approximately 6800 person-years, which can be considered a medium-scale study of secondary malignancy. Although the number of person-years was inferior to that in studies using large databases, the advantage of this study was that it provided highly accurate information on secondary malignancy by utilizing the characteristics of a single-center study. As in previous studies, our study validated the finding that RT after breast cancer surgery increases the risk of secondary malignancies in the Japanese population [2, 8, 9].

Several non-randomized studies have indicated that RT increases the risk of contralateral secondary breast cancer [15, 16]. In addition, Taylor et al. confirmed that postoperative breast cancer RT significantly increases the risk of contralateral breast cancer [8]. In support of these results, our study also implied that RT increased the RR of secondary breast cancer in the contralateral breast by 1.85-fold (95% CI 1.05–3.26); however, the increase was not significant (p = 0.053). This discrepancy is not simply attributed to the relatively small number of person-years but may be attributed to the longer follow-up period in our study. Taylor et al. also showed that the risk of contralateral breast cancer peaked at 5–9 years post-treatment and then declined as the observation period increased [8]. Accordingly, the incidence rate of contralateral breast cancer increased progressively up to 15 years after RT but was almost nonexistent thereafter (Fig. 1). Thus, the risk of contralateral breast cancer after RT for breast cancer should also consider the time lapse after the primary treatment.

Our study showed that RT for breast cancer increased the RR of secondary malignancies other than breast cancer by 2.71-fold among the patients compared with the general female population in Japan (95% CI 1.99–3.70, p < 0.001). Even when only malignancies detected at more than 10 years after RT were defined as secondary malignancies, the RR of secondary malignancies other than breast cancer was 1.91 (95% CI 1.33–2.73, p < 0.001). Previous meta-analyses demonstrated that the RR of secondary malignancies other than breast cancer was 1.22–1.23 [8, 9]. It should be noted that these studies seem to compare breast cancer patients with and without RT, whereas the present study compares breast cancer patients with the general population; thus, a simple comparison is not appropriate because of the different study methods used. The higher RR found may be related to other factors. There may be racial differences in secondary malignancies after breast cancer treatment, as reported by Calip et al. [10]. Besides, the genetic or biological attributes of each racial/ethnic group may explain the distribution of secondary cancer risk [17]. In line with this, Totoki et al. reported differences in genes encoding metabolic enzymes, including chromatin remodelers, in Asian and European ancestry populations [18]. In discussions of the risk of secondary malignancies, research focusing on such somatic mutations will become even more critical in future. Furthermore, socioeconomic, behavioral, and lifestyle factors may affect the risk of developing secondary malignancies. Further studies comprehensively including these factors are warranted to determine whether there is a difference in the risk of secondary malignancies between races.

Zablotska et al. reported an increased risk of lung and esophageal secondary cancers after breast cancer treatment; in both studies, only RT after mastectomy was significantly associated with increased risk [19, 20]. Our results are consistent with these findings. Morton et al. found that in female patients surviving breast cancer for more than 5 years, the excess odds ratio for secondary esophageal cancer increased linearly by 9% for each additional one Gy of tumor site dose [21]. Grantzau et al. reported that the rate of secondary lung cancer increased linearly, with 8.5% per delivered Gray to the lung, among breast cancer survivors for more than 5 years [22]. Therefore, it is likely that there is a dose-dependent increase in the risk of secondary cancer. Meanwhile, an increased incidence of secondary thyroid cancer was reported in patients with childhood cancer who received RT [23]. However, there was no excess risk of secondary cancers of the oral cavity and thyroid in the general population, which are relatively close to the irradiation fields in our study. Regarding secondary thyroid cancer, the risk may be higher in patients who received RT during childhood; however, there is probably no evidence that the risk of thyroid cancer increases as secondary cancer after RT in adults.

Herein, secondary malignancies at sites far from the irradiation field were also identified. The five patients with secondary ovarian cancer were less than 50 years old at the start of RT and may have had hereditary breast and ovarian cancer syndromes [24]. Lifestyle factors, such as alcohol consumption and smoking habits, and the effects of breast cancer chemotherapy on secondary malignancies in some patients should also be considered [25–27]. Thus, it is difficult to accurately investigate RT-induced secondary malignancies in a true sense. Best et al. reported that two variants on chromosome 6q21 were linked to secondary cancers in survivors of Hodgkin’s lymphoma treated with RT but not in adults [28]. Since there may be complex confounding of various factors in the development of secondary cancers, it would be desirable to use genetic mutations to assess the risk of cancer from radiation in future.

When the RT period was dichotomized (1961–1984 vs. 1984–2006), a higher incidence of secondary malignancies was observed in the group treated more recently (p < 0.001). Compared with conventional RT, IMRT increases the incidence rate of secondary cancers by 1.7-fold because it uses more treatment fields and exposes more normal tissue to low-dose radiation [29]. However, IMRT was not used in this study, and the reason for the higher incidence rate of secondary cancers in more recently treated patients was unclear. Technological advances in diagnostic medical devices may have led to the early detection of secondary cancers. Unfortunately, our study did not cover the details of the medical devices used to screen for secondary malignancies; therefore, further research that would consider this is necessary. As shown in Fig. 1, contralateral breast cancer appeared by approximately 20 years after RT, while the incidence rate of secondary malignancies other than contralateral breast cancer gradually increased over time. Accordingly, it is necessary to provide medical care and educate patients regarding the long-term development of secondary malignancies after RT for breast cancer.

Our study has several limitations. First, it was a single-center, retrospective study. This is a common problem in other studies on secondary malignancies, and prospective studies are needed to accurately identify the incidence rate of secondary malignancies. Second, we did not have any information on genetic factors, family history, adjuvant chemotherapy, or lifestyle factors that could influence the risk of secondary malignancies. Comprehensive studies that consider these factors are warranted to truly identify radiation-induced cancers. In addition, it would be necessary to mention the observation period. The median observation period in this study was 9.7 years, which is a moderate duration for a study of secondary malignancies. There was no significant difference in the occurrence of secondary malignancies when age was dichotomized by median age in this study. However, re-evaluation after long-term observation would be warranted because secondary malignancies still occur 10 years after treatment.

In conclusion, we found that the incidence of secondary malignancies after RT may be somewhat higher in Japanese patients with breast cancer than in the general population. Further large-scale studies are warranted to validate our findings.

## Data Availability

The datasets generated and/or analyzed during the current study are not publicly available due to institutional policy but are available from the corresponding author upon reasonable request.
